# Structured physical activity interventions as a complementary therapy for patients with inflammatory bowel disease – a scoping review and practical implications

**DOI:** 10.1186/s12876-019-1034-9

**Published:** 2019-07-02

**Authors:** Katharina G. Eckert, Isabelle Abbasi-Neureither, Maximilian Köppel, Gerhard Huber

**Affiliations:** 1 IST University of Applied Sciences, Health Management and Public Health, Düsseldorf, Germany; 2 Practice for Internal Medicine and Gastroenterology, Heidelberg, Germany; 3 Penn State University, Department of Public Health Science, Hershey, USA; 4 University of Heidelberg, Institute of sport and sportscience, Heidelberg, Germany

**Keywords:** Inflammatory bowel disease, Physical activity intervention, Disease activity, Disease management

## Abstract

**Background:**

Patients with Inflammatory Bowel Disease (IBD) also suffer from a wide range of additional disorders, which may be caused by the disease, the side effect of the medication, or a lack of physical activity (PA). This results in reduced physical and psychological wellbeing. However, as known from other chronic diseases exercise could be utilized as supportive therapy for IBD patients. Main goals of this article are (a) collecting data of the effects structured physical activity interventions have on validated clinical parameters of IBD and health related symptoms, (b) developing activity recommendations for this clientele.

**Methods:**

A scoping review was conducted, searching for relevant articles published until May 2018, which investigated the effects of structured exercise interventions in IBD patients. The heterogeneity of the outcomes and the interventions did not support a quantitative synthesis thus, a qualitative discussion of the studies is provided.

**Results:**

After reviewing 353 records, 13 eligible articles were identified. Five studies investigated aerobic exercise, three studies resistance exercise, three studies mind-body therapies and two studies yoga. The quality of the studies is mixed, and the duration is rather short for exercise interventions. Only few studies assessed validated IBD activity markers or inflammatory biomarkers. Nevertheless, the patients showed an increase in fitness, bone mineral density (BMD), quality of life and a decrease of IBD induced stress and anxiety. No severe adversial events were reported.

**Conclusion:**

Even though the evidence is limited the application of exercise interventions in IBD patients can be assumed to be safe and beneficial for the patients‘ overall-health, and IBD specific physical and psychosocial symptoms. But there is still a high demand for more thoroughly conducted studies, focussing on important clinical outcome parameters.

## Background

Inflammatory Bowel Disease (IBD) is a chronic intestinal inflammatory illness, characterized by periods of remissions and relapses [1, 2]. The two main types of IBD are Crohn’s disease (CD) and ulcerative colitis (UC). Whereas UC is restricted to the large intestines and the rectum, specifically the mucosa and superficial submucosa, CD is transmural and can affect any part of the gastrointestinal tract [3, 4]. Until now the highest relative prevalence of IBD has been reported in European countries (UC, 505 per 100.000 persons in Norway; CD, 322 per 100.000 persons in Germany) in addition it appears be rising worldwide. While most of the industrialized nations display stable incidence rates of IBD, an increasing number of new cases can be observed in emerging countries [5].

There is still a lack of knowledge in regards to the pathophysiology and the etiology of IBD, meaning, its origin seems to be multifactorial and influenced by an abnormal immune response to gut microbes in a genetically predisposed person [6, 7]. Both diseases show symptoms like diarrhea, abdominal cramps and pain, rectal bleeding and weight loss [8]. These symptoms are often accompanied by further extraintestinal health problems [9], such as reduced BMD and osteoporosis [10–12] fatigue [13, 14], or depressive symptoms [15], a decrease in physical fitness [16–18], and an impaired overall health related quality of life (HrQoL) [12, 19, 20] that are caused by the disease, as side effects of the medical treatments [21, 22] and a lack of PA [23, 24].

Medical treatment is often necessary throughout the patient’s lifetime. Current treatments for IBD consist of medications like 5-aminosalicylic acid compounds, corticosteroids, immunosuppressants and biologics. The main cost driver is Anti-Tumor Necrosis Factor alpha (Anti-TNF α) therapy, which accounts for 64% of the total costs in CD and 31% of the total costs in UC [25]. Studies have shown that the total annual direct costs of patients with IBD (i.e. medication, diagnostics, physicians and other healthcare services) range from $ 11 to $ 28 billion in the United States [26] and from 4.6 € to 5.6 billion € in Europe [27]. This data implicates that IBD represents a challenge for health care systems globally. The main goal in the treatment of IBD is to reduce the quantity and the quality of the inflammation, ideally resulting in a clinical remission. Disease activity is quantified via three classes of indicators: clinical symptoms, inflammatory markers and HrQoL [28].

Since there is no cure for IBD yet, currently available drugs can only tackle its symptoms by inhibiting inflammation and delaying relapses. Despite their efficiency to relief symptoms, undesirable side effects are common [21, 22]. Therefore, many patients look for supportive and adjunctive therapies. PA has proven to be a factor in helping other chronic conditions, thus, it could be a feasible approach for IBD patients [29]. However, not enough is known about the impact of structured PA interventions on the clinical course of IBD or side effects associated with the disease and its treatments [30]. Hence, these are the main goals of this paper:

- Collecting data and giving evidence about the effects of structured physical exercise interventions on inflammatory biomarkers, clinical activity metrics and HrQoL as directly with the disease associated primary outcomes, and on physical and psychosocial functions (e.g. muscle performance, BMD, cardiorespiratory fitness, fatigue) as secondary outcomes.
- Developing PA recommendations, which are specific for IBD patients.

## Methods

### Search strategy

In May 2018 a literature search was conducted using the databases of PubMed, Cinhal and Web of Science for relevant abstracts and articles on IBD and PA interventions. The search terms included: exercise, physical activity, PA, motor activity, resistance training, strength training, endurance training, aerobic, sport for the semantic space of exercise. These terms were paired with inflammatory bowel disease, IBD, Crohn’s disease, CD, ulcerative colitis, UC (using AND as operator). By screening Google Scholar and the reference lists of the included studies, overlooked studies were added. All identified titles and abstracts were assessed for eligibility by two reviewers independently (KE and MK).

### Study selection

The following criteria determined which references were included: Only adults (≥18 years) diagnosed either with CD or UC. Only full-text articles written in English were selected. Only longitudinal study designs with PA interventions were included. Studies were not limited with regards to type of PA. All publication years were included in the search. Studies on Irritable Bowel Syndrome were excluded.

### Data extraction

Included full texts were assessed by KE and GH independently. The heterogeneity of the conducted interventions, the inconsistency in outcome measures and the complex nature of IBD (phenotype, location, disease activity, medication and disease related symptoms) made a meta-analytical synthesis of the outcomes implausible, thus a qualitative approach was preferred to present the current evidence. All eligible studies were classified according to 2011 Oxford Centre for Evidence-Based Medicine (OCEBM) levels of evidence (first column: Does this intervention help?) [31]. The review was structured according to the Preferred Reporting Items for Systematic Reviews and Meta-Analyses (the PRISMA statement) [32].

## Results

**Fig. 1:**
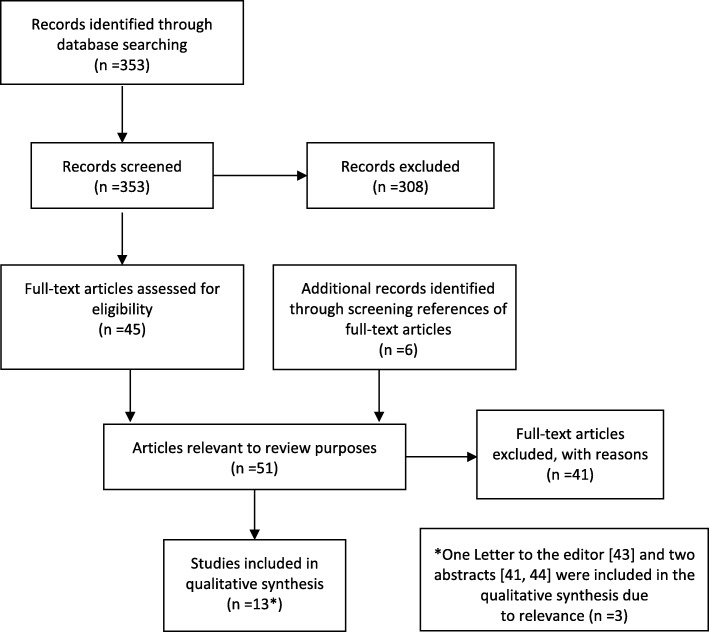
Flow diagram of literature search and study selection

A total of *n* = 353 articles were identified. After excluding duplicates and scanning titles as well as abstracts, *n* = 45 articles were retrieved and examined. Another *n* = 6 articles were added after scanning the references of chosen articles. These *n* = 51 articles were eligible for full text screening, resulting in further exclusion of *n* = 41 articles. Although two abstracts and one letter to the editor were excluded after title and abstract screening, we included them after all due to their relevance. Thus, 13 articles were used for the qualitative analysis (Fig. 1). Reasons for the exclusion of articles are presented in Table 1.

**Table 1:** Reasons for exclusion of full-text articles

Reason for exclusion	Number of articles excluded
No PA intervention	*n*=29
No IBD patients	*n*=5
Study protocol	*n*=1
Use of same study sample	*n*=2
Reviews / no Interventions	*n*=4
Total number of articles excluded after full text screening	***n*** **=41**

### Characteristics of the studies

Thirteen studies were included in this review. Seven studies were randomized controlled trials (RCT) [7, 30, 33–37] and six studies used a quasi-experimental design [38–43].

Selected studies were conducted between the years 1999 and 2017, with *n* = 6 published within the last 4 years. The sample sizes vary from 6 to 107 subjects. Five of the studies included patients with CD only two investigated patients with UC and six studies incorporated both diseases. All study participants were either in remission or had a mild to moderate disease activity at the time of intervention. With regards to methodological limitations, the study results are presented descriptively in accordance with their primary results. Statistical parameters are listed if they were provided. (Table 2).

**Table 2:** Qualitative synthesis of studies examining the effects of structured exercise interventions in patients with IBD

Author	Subjects and design	Endpoints	Medication EG (CG)	Methods	Duration and frequency	Main findings	Adverse events	Level of evidence
Robinson et al., [33]	107 patients with CD, mild to moderate disease activity; block randomization (EG: n=53, CG: n=54)	BMD	All under steroid use	Home-based; floor-based, progressive low-impact dynamic resistance training	At least twice a week, with a min. of 10 sessions per month; 1 year	Fully compliant patients (14): BMD increased at the femoral neck (n.s.), the spine (n.s.), the Ward’s triangle (n.s.) and the trochanter major ([EG-CG] (95% CI) = 4.67 (0.86-8.48), p=.02)	Not reported	2
D’Inca et al., [38]	6 CD patients in remission; 6 healthy controls	Disease activity, various gastrointestinal parameters	Not reported	Cycling exercise	Cycle ergometer exercise at 60% of max. oxygen intake; once for 1 hour	No statistically significant effects on gastrointestinal parameters; no change in disease activity	None	3
Loudon et al., [39]	12 physically inactive patients with inactive or mildly active CD, no controls	Stress Index, HrQoL, disease activity, fitness, BMI	Prednisone n=4; 5-ASA n=5; 6-MP n=6; no medication n=2	Supervised and unsupervised walking program (indoor track)	3 sessions a week (20-35 min); 12 weeks	Significant improvements in IBD Stress Index (mean change study outset (29.2±15.4) to completion (19.5±10.8) p<.001), IBDQ (172±27 to 189±12, p=.01), HBI (5.9±5.0 to 3.6±3.1, p=.02), VO_2_max (30.6±4.7 to 32.4±4.8, p<.01), BMI (24.3±5.3 to 23.9±5.3, p=0.07)	None	4
Candow et al., [40]	12 CD patients, no controls; disease activity not specified	Disease activity, muscle strength	Not reported	Supervised resistance training (12 exercises)	3 times a week over the course of 12 weeks, 3 sets, 8-10 repetitions; 60-70% of 1RM	Significant increase in muscle strength (p<.05); no change in disease activity (HBI)	None	4
Elsenbruch et al., [34]	30 UC patients in remission or low disease activity; randomized controlled trial	Neuroendocrine and cellular immune parameters, HrQoL, disease activity	5-ASA n=8 (7); probiotics n=1 (3); ironsulfate n=0 (1) no medication n=6 (4)	Structured and supervised mind-body therapy (includes stress management training, moderate exercise, Mediterranean diet, cognitive behavioral techniques with focus on self-care strategies)	60-hour program over a 10-week period (i.e. 6 h on 1 day every week)	Significant improvements in HrQoL (SF-36 short: psychosocial health sum score p<.05, mean change EG=7.2±10.7; mean change CG = 0.0±8.5) and IBDQ (bowel symptoms: d=0.52, p<.01); no statistically significant group differences in lymphocyte sub-set numbers or production of TNF α and RI	Not reported	2
Gupta et al., [41]	175 patients with different chronic conditions (n=18 with gastrointestinal problems including CD, disease activity not specified)	Anxiety scores	Not specified	Lifestyle intervention	Yoga, breathing exercise, mediation, stress management and nutrition education; 5+3 days with a two day break for weekend	No statistically significant change in anxiety levels (STAI)	Not reported	4
Ng et al., [30]	32 patients in remission or with mildly active CD, matched and randomized	HrQoL, disease activity, Stress Index	5-ASA n=6 (6); no medication n=10 (10)	Independant walking program	60% HRmax during exercise, 3 times a week over 3 month; 30 min per session	Significant improvements in IDB Stress Index (p<.05), disease related dysfunction (IBDQ) (p<.05) and reduction in HBI (p<.01)	None	2
De Souza Tajiri et al., [42]	19 patients (CD: n=10, UC: n=9), no controls; disease activity not specified	Thigh circumference, bodyweight, quadriceps strength, HrQoL	Not reported	Progressive resistance training	Knee extension; first 4 weeks: 50% 1RM, 3 sets of 12 repetitions; last 4 weeks: weekly increase of load by 10% until 80% of max. load	Significant improvements in quadriceps strength (greater than 40%, p<.001), IBDQ (mean changes baseline 156.3±29.0 to post 180.5±24.2, p<.001). No statistically significant changes in thigh circumference and bodyweight	None	4
Gerbarg et al., [36]	25 patients with mild to moderate IBD, randomized	Psychological and physical symptoms (HrQoL), inflammatory markers	No medication n=5; all other mixed medications (biologics, immunosuppressive; corticosteroids; mesalamines)	EG: 9 hours administered Breath-Body-Mind Workshop (BBMW) (breathing, Qigong, mediation) CG: 9 hours educational seminar (ES) (information about IBD and its treatment)	EG: BBMW and 26 weeks homebased, self-administered sessions, every day for 20 min	No between group differences IBDQ (mean change EG= 12.57±15.85, mean change CG= -1.73±19.91; p=.08); Significant changes in CRP (median change EG: baseline 1026.0 to post 730.0; p=.01; median change CG: 8590.0 to 7180.0, p=.39) but not in FCP (median change EG: baseline 216.3 to post 155.9, p=.78; median change CG: 157.8 to 341.5, p=.59),	None	2
Klare et al., [37]	30 patients with mild to moderate IBD, randomized controlled trial	HrQoL, disease activity, BMI	Prednisolone n=4 (1); budesonide n=3 (2); mesalazine n=3 (5); ASA/5-MP n=3 (5)	Supervised outdoor running program for untrained people	Moderate intensity, equated by BMI; 3 times a week for 10 weeks	Significant improvements of IBDQ social dimension ([EG-CG] (95% CI) = 4.4 (0.6-8.2), p=.03); no changes in disease activity (CDAI: [EG-CD] (95% CI) = -3.7 (-35.8-29.3, p=.81; RI: [EG-CG] (95% CI) = -0.2 (-2,6-2.3), p=.88); BMI ([EG-CG] (95% CI) = 0.4 (0.0-0.9), p=.08) or laboratory results (Lc: [EG-CG] (95% CI) = -0.7(-2.3-0.9), p=.39; CRP: [EG-CG] (95% CI) = 0.0 (-0.3-0.2), p=.88; FCP: [EG-CG] (95% CI) = -25.3 (-433.6-383.0), p=.90	None	2
Sharma et al., [7]	87 patients (CD: n=36, UC: n=51) in clinical remission, randomly allocated to EG or CG	Stress Index, anxiety, cardiovascular autonomic functions, immune markers	“all treated with maintenance dose of mesalamines and azathriopine” (p.103)	Supervised Yoga intervention (physical postures, pranayama, meditation)	1 hour a day for 8 weeks	No statistically significant group differences in any outcome parameter (overall), but significant differences within the UC groups (EG and CG) in State (mean change baseline from 38.9±8.9 to post 32.8±8.2, p=.01) and Trait (mean change from 49.5±8.7 to 41.2±8.2, p=.001) anxiety levels (STAI); fewer UC patients reported arthralgia (p<.05)	Not reported	2
Hassid et al., [43]	10 patients (CD n=7, UC n=3), no controls; disease activity not specified	Disease activity	Not reported	Different types of intensive exercise: marathon (1), half-marathon (5), long bicycle ride (>45 miles) (3), triathlon (1)	Once	No statistically significant change in disease activity (HBI and SCCAI); no abnormally elevated FCP	None	4
Cramer et al., [35]	77 UC patients, randomly assigned; in remission	HrQoL, disease activity	Biologics n=4 (6); immunosuppressive n=0 (1); thiopurines n=10 (10); mesalazine n=30 (28); probiotics n=5 (1)	Supervised traditional hatha yoga intervention (EG); two self-care books - without instructions for using - providing general information on UC (CG)	90 min weekly over a period of 12 weeks	Significant increase of HrQoL after 12 weeks (IBDQ: [EG-CG] (95% CI) = 14.7 (2.4-26.9), p=.02) and after 24 weeks ([EG-CG] (95% CI) = 16.4 (2.5-30.3), p=.02); disease activity (RI: [EG-CG] (95% CI) = -1.2 (-0.1-[-2.3]), p=.03)	None	2

*5-ASA*: 5-Aminosalicylic Acid; *6-MP*: 6-Mercatopurine; BMD: Bone mineral density; *BMI*: body mass index; *BBMW*: Breath-Body-Mind Workshop; *CD*: Crohn’s Disease; *CDAI*: Crohn’s Disease Activity Index; *CG*: Control Group; *EG*: Experimental Group; *FCP*: fecal calprotectin; *HBI*: Harvey and Bradshaw Index; *HrQoL*: Health related Quality of Life; *IBD*: Inflammatory Bowel Disease; *IBDQ*: Inflammatory Bowel Disease Questionnaire; *Lc*: Leucocyte count; *min*: minutes; *n.s*.: not significant; *1RM*: One-Repetition-Maximum; *RI*: Rachmilewitz Index; *SCCAI*: Simple Clinical Colitis Activity index; *STAI*: State and Trait Anxiety Inventory; *UC*: Ulcerative Colitis

### Findings by type of exercise intervention

#### Cardiovascular training

Five studies reported the effects of a cardiovascular training on diverse health outcomes. D’Inca and colleagues found neither statistically significant changes in the assessed gastrointestinal health parameters nor significant changes in disease activity after 1 hour of cycle ergometer exercise [38]. On the other hand, Loudon et al. [39] and Ng et al. [30] observed positive effects on stress parameters (IBD Stress Index) *p* < .001 [39] and *p* < .05 [30]), HrQoL (IBD Questionnaire (IBDQ) *p* = .01 [39] and p < .05 [30]) and cardiorespiratory fitness (VO_2_max *p* < .01 [39]) in people with CD, involving a walking intervention taking place once a week for 3 months. A supervised outdoor running program, three times a week for 10 weeks, resulted in significant improvements in the social dimension of HrQoL (IBDQ social dimension *p* = .03) but showed no changes in disease activity (Crohn’s Disease Activity Index (CDAI) *p* = .81; Rachmilewitz Index (RI) *p* = .88) or laboratory parameters (Leucocyte count (Lc) *p* = .39, C-reactive protein (CRP) p = .88, fecal calprotectin (FCP) *p* = .90) [36]. Hassid et al. examined the effects of different types of high voluminous sport activities (marathon, half-marathon, long bicycle rides and triathlon) on disease activity (Harvey and Bradshaw Index (HBI); Simple Clinical Colitis Activity Index (SCCAI)) in 17 mixed IBD patients. As result, no significant changes or harmful effects were discovered instantly and a week after the exercise [43].

#### Strength training

In three studies, the patients participated strength training, with two supervised interventions were executed on weightlifting machines [40, 42]. With only one study assessing a disease activity index [40]. Candow and colleagues chose a 12-week intervention on 12 different training machines with constant intensity and number of repetitions [40]. The participants in De Souza Tajiris et al. study performed an isolated, progressive training involving only the quadriceps muscle [42]. Both found a significant increase in muscle strength (*p* < .05 [40], *p* < .001 [42]). Nevertheless, the progressive training protocol leads to a significant improvement in patients’ HrQoL (IBDQ p < .001 [42]), but failed to decrease the disease activity (HBI) significantly [40]. In one study, a home- and floor-based low impact dynamic resistance training was conducted. The intervention lasted for over 1 year and had improvements in BMD as primary outcome. Fully compliant patients showed a significant increase in BMD for the trochanter major (*p* = .02). All other assessed anatomical structures showed no statistically significant increase in BMD [33].

#### Yoga

Cramer et al. randomly assigned 77 UC patients to either a 12-weeks lasting traditional Hatha yoga group or an inactive control group. Participants of the yoga group showed significantly higher HrQoL scores (IBDQ *p* = .02) and lower disease activity (RI *p* = .03) 24 weeks after the intervention [37]. Sharma et al. carried out a supervised yoga intervention over the course of 8 weeks with patients in remission. While patients with UC showed slightly improved anxiety scores (STAI: State (*p* = .01) and Trait (*p* = .001) anxiety levels) and fewer arthralgia (*p* < .05), patients with CD displayed no changes. Also no impact on cardiovascular autonomic functions or immune markers (Lc, Serum eosinophilic cationic protein (ECP)) was observed [7].

#### Lifestyle intervention – mixed methods

In three studies various mind-body therapies were conducted, including e.g. yoga, meditation, stress management, nutrition, relaxation techniques, education about the disease and cognitive behavioral techniques. Gupta et al. found no effects on anxiety levels, disease activity or inflammatory markers while Gerbarg and colleagues detected significant improvements in CRP for the EG (*p* = .01) but not for the CG (*p* = .39) [36, 41]. In a structured and supervised mind-body therapy over a 10-week period (6 h on 1 day per week), higher HrQoL scores for the EG were observed (SF-36 psychosocial health sum *p* < .05 and IBDQ *p* < .01), but no effects on disease activity (RI) or neuroendocrine and cellular immune measures (Lc,TNF α) [34].

## Discussion

The current review investigated the effects of structured PA interventions on inflammatory biomarkers, clinical activity indices and HrQoL as directly with the disease associated primary outcomes and of physical and psychosocial functions in patients as secondary outcomes. It follows a presentation of the biological, physical and psychosocial effects. Additionally, the known effects and mechanisms of exercise in other clinical populations [29, 44] will be displayed and their potential for IBD patients discussed.

### Biologic rationale for benefits of PA interventions in IBDs

#### a) Inflammatory biomarkers

The conducted exercise trials report only minor changes in laboratory markers [7, 34, 36, 37]. Three of the included studies investigated the effects of exercise on inflammatory biomarkers like FCP, CRP, Lc and TNF α, with merely one study showing a significant reduction in CRP [36]. Despite the importance of TNF α in IBD, only one study explored this parameter; however, no significant effect on the TNF α basal levels were observed within the treatment group, that conducted mind-body therapy [34]. To the best of our knowledge, only one additional study has investigated the relationship of PA and inflammation in IBD patients [54]. Plöger et al. reported a positive association of exercise and increased levels of Interleukin 6 (IL-6) in children with IBD. However this study was not included in this review, since it is limited to studies conducted in adults.

The anti-inflammatory effect of PA is well investigated in Type 2 Diabetes and Cardiovascular diseases [45]. It can be attributed to the release of cytokines, such as IL-6, which affects the systemic inflammation via crosstalk on TNF α [46, 47]. Observations of anti-inflammatory pathways were made in murine models and have also been confirmed in exercise studies with humans, involving strength training [48] and strenuous aerobic exercise [49]. Even though the evidence provides a framework for similar effects in IBD patients, it is not possible to draw clear conclusions from the analyzed studies. However, the evidence with regards to the beneficial effects of moderate PA on the immune system seems promising [55, 56]. This is particularly important since TNF α is a major pathological marker in IBD [50], while the serum level of TNF α correlates with the clinical activity of CD and UC [51].

The characteristics of the exercise stimulus, such as frequency, intensity, time (duration) and type (FITT-Criteria) [52], have a direct impact on the release of the anti-inflammatory cytokines. The IL-6 plasma concentration increases almost exponentially in relation to the duration of exercise. For instance, 30 min of endurance training at 75% VO_2_max increases the IL-6 plasma concentration by five times. Further investigations have shown that the type of exercise as well as the amount of recruited muscle mass determine the extent of IL-6 release [53]. PA involving several large muscle groups, such as brisk walking or rowing, has shown the highest plasma IL-6 increase [45]. Although other mediators, like adrenaline, that blunts the appearance of TNF α, may contribute to the anti-inflammatory effect of exercise, the findings from Pedersen and colleagues emphasize that muscle derived IL-6 is the true anti-inflammatory exercise factor [53].

#### Disease activity

Eight out of the 13 included studies assessed changes in disease activity by using validated metrics [30, 34, 35, 37–40, 43] (HBI *n* = 4, CDAI *n* = 2; RI n = 2, SCCAI *n* = 1). In three of these studies, positive effects on the disease activity indices were found. The five remaining studies found neither positive nor negative effects of the interventions on clinical disease activity. Given that the studies did not find any increase of the disease activity, exercise can be seen as feasible and safe for this population. To further investigate potential mechanisms and effects of exercise on the disease activity, it might be useful to look into the single disease activity parameters of the assessment tools, considering a physiological rationale.

### Health benefits of PA interventions in IBD

#### a) Physical effects

The included interventional studies barely report physical effects thus estimations of the cardiovascular, muscular, and bony adaptions remain uncertain and had to be derived from studies conducted in other clinical populations [29]. Nevertheless, the few studies that investigated the effect of the interventions on physical parameters show an increase in VO_2_max [39], muscle strength [40, 42] and BMD [33].

An increased risk to develop osteopenia or osteoporosis, is very common in IBD. Approximately 40% of all IBD patients have developed osteoporosis [10–12]. Nonetheless, the causes of low BMD are multifactoral in IBD patients, an increase in pro-inflammatory cytokines disturbes the bone remodelling process by increasing osteoclastic and inhibiting osteoblastic functions [10, 57]. Most IBD patients present a decreased muscle mass in comparison to their healthy controls [16], which can be attributed to the catabolic effect of cytokines [58, 59], physical inactivity [23, 24], malnutrition and malabsorption [60] as well as the use of corticosteroids [17, 61]. IBD patients also seem to suffer from reduced cardiorespiratory fitness, with a direct link to low PA levels [16, 18]. This is of significance, since reduced physical fitness levels are correlated to increased systemic inflammation [47].

Muscle strength, BMD as well as VO_2_max are essential predictors of all-cause mortality in a wide range of populations [62], thus, these can be seen as surrogate parameters for several health parameters, such as physical functioning and autonomy.

#### b) Psychological effects

Although fatigue affects nearly 50% of patients in clinical remission and over 80% with active disease [65], none of the included studies assessed it. Fatigue, an intense tiredness with reduced energy levels and feelings of exhaustion [63], is considered to be the most burdensome symptom in IBD patients [14, 64]. As known from research of other chronically ill people, PA can effectively address the symptoms of fatigue [65–67]. Fatigue contributes to a loss in muscle mass due to the lack of PA [23, 24, 68] especially in a population with an existing muscular disadvantaged [16]. Hitherto, the etiology of fatigue cannot be explained [65], though, an assessment of Cancer Related Fatigue (CRF) shows, that the amount of circulating pro-inflammatory blood cytokines are strongly associated with the severity of CRF [69–71]. Although studies involving IBD patients do not investigate fatigue, studies with CRF patients seem to include rather promising information [71], meaning the modes of action PA exhibits on CRF may be beneficial for IBD patients [65, 67].

Stress and anxiety are typically perceived by IBD patients and can trigger relapses [72]. One of the two studies assessed anxiety showed significant reductions in both, the State and the Trait sum scores [7]. In two out of three studies, the aerobic exercise groups showed a significant reduction in stress [30, 39]. Excercise may be a useful treatment for anxiety and stress. Nevertheless the relative paucity of evidence leaves the question open whether a direct mechanism for exercise to reduce anxiey exists [73]. Possible explanations of reduced anxiety levels after PA interventions can be attributed to improved health-related self-efficacy [74] and perceived control [75] through fitness gains. Both are known to positively influence adherence to treatment and medication [76]. These potential effects of PA are essential for all chronically ill patients, and thus for people with IBD.

Having a chronic disease that affects bowel function can impact many aspects of life beyond the medical symptoms, e.g. social life and the patients’ HrQoL. The ultimate target of therapy for IBD is to alter the course of the disease and thus improve the quality of life. The HrQoL of IBD patients is determined by the markers of the disease activity, such as increased clinical activity markers, number of relapses, working disabilities and hospitalization [77]. Research shows consistent associations between moderate PA with increased HrQoL not only for healthy individuals [78] but also for chronically ill people [79, 80]. Six of the studies report similar effects on the general HrQoL as well as on the HrQoL-subscales for structured PA interventions in IBD [30, 34, 35, 37, 39, 42]. A recent cross-sectional study found that engaging in higher quantities of walking in a moderate intensity (equivalent to recommended activity guidelines) is independently associated with increased physical HrQoL in people with IBD [81]. PA may be an efficient non-pharmacological supportive treatment in IBD, and will increase patient’s HrQoL, but lack of data from rigorous, methodologically sound RCTs precludes any definitive conclusions about its effectiveness.

In summary structured PA interventions may have the potential to break the cycle of physical inactivity, muscular deconditioning, poor fitness and decreased BMD, leading to reduced fatigue and anxiety, which ultimately improve stress, disease management abilities and HrQoL. Besides these effects it is possible that regular PA and exercise have a beneficial anti-inflammatory impact on immunologic biomarkers (e.g. CRP, TNF α) in IBD. In any event, exercise can improve the physical and psychological health of the patients.

**Fig. 2:**
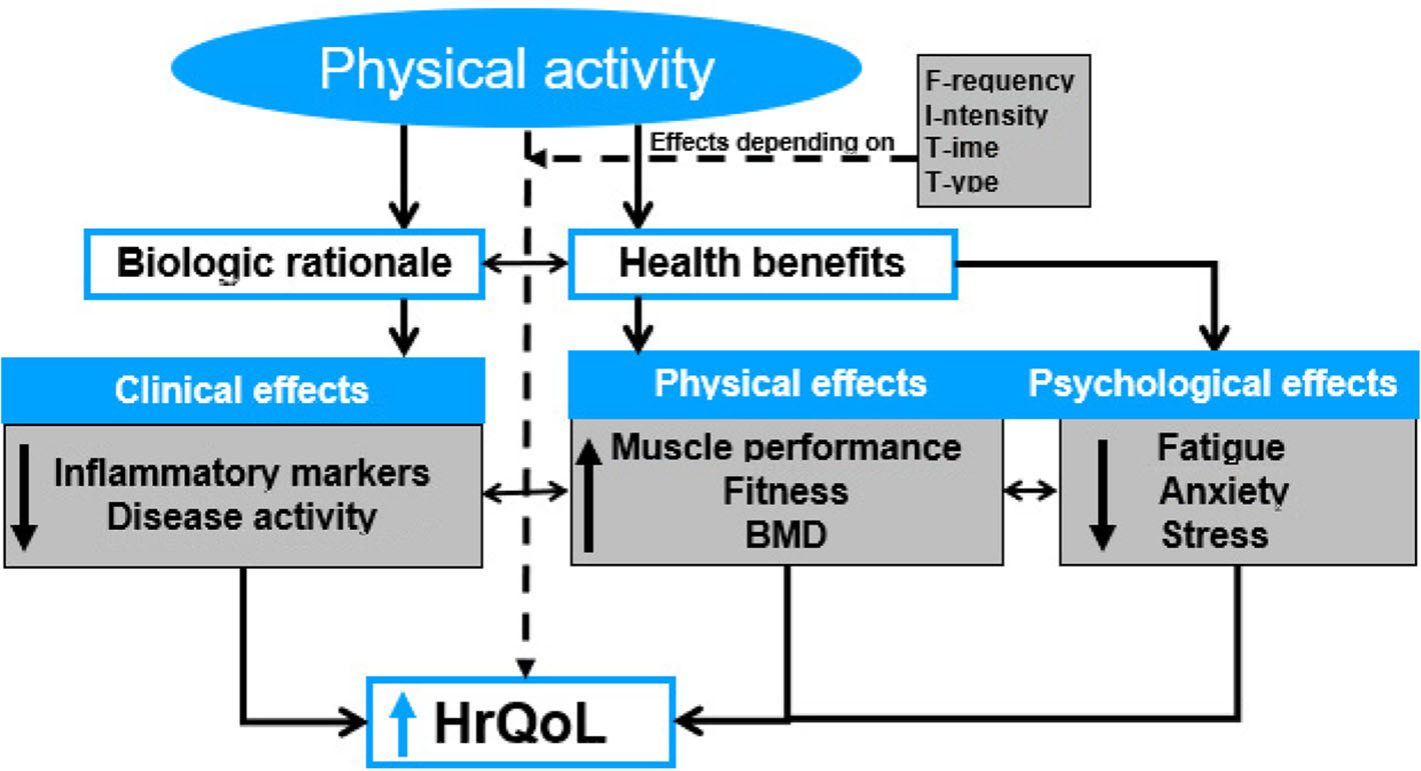
Possible clinical, physical and psychological effects of PA in IB

Most of the existing studies have significant methodological limitations. In detail we acknowledge the following: As known, the recruitment of patients for exercise studies via advertisement, e.g., newspaper, radio, flyer or email, results in an ascertainment bias, i.e. only those who already have a high motivation to exercise will participate. Due to the recruitment pathways it is possible to only reach certain populations, which can skew the distribution of socioeconomic and educational variables. The studies were limited to IBD patients with mild to moderate disease severity, which narrows the applicability of the results. In addition, the initial PA level was not assessed. Changes in PA levels during the study are a crucial aspect since they impact the benefits from exercise. None of the studies conducted an a priori power analysis, thus all studies, except of Sharma et al. [7] and Cramer et al. [35], had very small sample sizes that lead to uncertain parameter estimates. Merely one study reported blinding of the control group. Since all studies reported every measured outcome and results do not seem to support the interventions, a publication bias seems unlikely, but we acknowledge that the absence of a research librarian during the reviewing process may limit our results. Because of the limited data available, mechanisms of exercise used for IBD can only be hypothesized at this time (Fig. 2).

## Conclusion

As stated by Pedersen et al., PA ‘...represents a natural, strong anti-inflammatory and metabolic-improving strategy with minor side effects … [45] p.607]’. The benefits of structured PA interventions on IBD symptoms have not been sufficiently investigated up to now [82], even though they are evident in other chronic diseases [29]. Nevertheless, low to moderate PA seems safe for patients with mild to moderate IBD. Only two out of 13 studies reported slightly negative effects immediately after the PA intervention, though they did not persist [43, 35].

We assume, that the little impact of the interventions on health parameters might have been caused by the heterogenous training methods and chosen intensities. Further research upon the most appropriate type of exercise is required.

Conclusively we deviate - based on this review and derived from existing evidence of morphologic adaptations and psychosocial effects of PA in other populations - PA recommendations for IBD patients along with methodological recommendations for upcoming studies:

- Type and volume of exercise must be sufficient to stimulate anti-inflammatory effects. Research has shown, that the anti-inflammatory output of exercise depends on the FITT-Criteria [45] as well as the amount of recruited muscle mass [53]. In order to assist clinicians to advise patients better and improve the design of future exercise studies, a representation of recommended FITT criteria, has been provided in Table 3.
- The maintenance of an active lifestyle is the main goal of all exercise interventions. Thus, future studies need to implement psychological techniques to enhance motivation, self-regulation and self-reflection, in order to enable patients implement PA for their individual disease management [83].
- An agreement in standardized assessment instruments including the most important health outcomes in IBD would be necessary for the quantitative synthesis of future studies [28, 84]. The interventions as well as outcome assessments should be multidimensional, i.e. the combination of disease-specific clinical markers with physiological and psychological parameters.
- Future investigations should assess quality of life as it is an important aspect of medical decision-making and a major goal of therapy.
- Because of the cyclical nature of IBD and the individuality of the disease patterns, it is difficult to conduct standardized large-scale RCTs. To counteract this methodological obstacle the application of systematic N-of-1-trials can be helpful [85].

**Table 3:** Physical activity recommendations for patients with mild-to-moderate IBD

*FITT-Criteria*	*The patient should….*
F-requency	...engage in moderate PA at least three times a week, even better five times a week.
I-ntensity	... choose an activity which increases the energy expenditure by at least a factor of three or four, as it is the case for brisk walking. For exercise control via heart rate (HR), the intensity of the exercise should be between 60 – 80% of the maximum HR. Bear in mind: moderate intensity is key in order to improve inflammation.
T-ime	… exercise for at least 30 minutes per day (more is even better, if tolerated).
T-ype	... engage in an enjoyable activity, to increase the probability to maintain this behavior. Exercising in groups can increase the motivation. A mixture of endurance and resistance exercise is favorable, because it avoids unilateral training and emphasizes the use of all big muscle groups. ... increase the amount of leisure time PA. “Walk before you run”.

## Data Availability

Not applicable.

## Abbreviations

Anti-TNF α: Anti-Tumor Necrosis Factor alpha
BMD: Bone mineral density
BMI: Body mass index
CD: Crohn’s Disease
CG: Control Group
CRF: Cancer Related Fatigue
CRP: C-reactive protein
ECP: Serum eosinophilic cationic protein
EG: Experimental Group
FCP: Fecal calprotectin
HR: Heart rate
HrQoL: Health related Quality of Life
IBD: Stress Index: Inflammatory Bowel Disease Stress Index
IBD: Inflammatory Bowel Disease
IBDQ: Inflammatory Bowel Disease Questionnaire
IL-6: Interleukin 6
Lc: Leucocyte count
min: minutes
n.s: not significant
OCEBM: Oxford Center for Evidence-Based Medicine
PA: Physical activity
PRISMA: Preferred Reporting Items for Systematic Reviews and Meta-Analyses
RCT: Randomized Controlled Trail
RI: Rachmilewitz Index
SCCAI: Simple Clinical Colitis Activity Index
STAI: State and Trait Anxiety Levels
TNF α: Tumor Necrosis Factor α
UC: Ulcerative colitis
